# Role of KLF4 and SIAT7A interaction accelerates myocardial hypertrophy induced by Ang II

**DOI:** 10.1111/jcmm.70144

**Published:** 2024-10-21

**Authors:** Qiying Yao, Xinrui Hu, Tiantian Bian, Qing Zhang, Zhao Xue, Yuesheng Lv, Shupeng Ren, Yue Chen, Dongmei Zhang, Liang Chen

**Affiliations:** ^1^ Department of Physiology Dalian Medical University Dalian Liaoning China; ^2^ Department of Cardiology The Second Affiliated Hospital of Dalian Medical University Dalian China; ^3^ Institute of Cancer Stem Cell Dalian Medical University Dalian China

**Keywords:** angiotensin II, KLF4, myocardial hypertrophy, Sialyl‐Tn, SIAT7A

## Abstract

Sialylation catalysed by sialyltransferase 7A (SIAT7A) plays a role in the development of cardiac hypertrophy. However, the regulatory mechanisms upstream of SIAT7A in this context remain poorly elucidated. Previous study demonstrated that KLF4 activates the *SIAT7A* gene in ischemic myocardium by binding to its promoter region. Nevertheless, the potential involvement of KLF4 in regulating SIAT7A expression in Ang II‐induced hypertrophic cardiomyocytes remains uncertain. This study seeks to deepen the underlying mechanisms of the KLF4 and SIAT7A interaction in the progression of Ang II‐induced cardiac hypertrophy. The results showed a concurrent increase in SIAT7A and KLF4 levels in hypertrophic myocardium of essential hypertension patients and in hypertrophic cardiomyocytes stimulated by Ang II. In vitro experiments revealed that reducing KLF4 levels led to a decrease in both SIAT7A synthesis and Sialyl‐Tn antigen expression, consequently inhibiting Ang II‐induced cardiomyocyte hypertrophy. Intriguingly, reducing SIAT7A levels also resulted in decreased KLF4 expression and suppression cardiomyocyte hypertrophy. Consistent with this, elevating SIAT7A levels increased KLF4 expression and exacerbated cardiomyocyte hypertrophy in both in vivo and in vitro experiments. Additionally, a time‐course analysis indicated that KLF4 expression preceded that of SIAT7A. Luciferase reporter assays further confirmed that modulating *SIAT7A* levels directly influenced the transcriptional activity of *KLF4* in cardiomyocytes. In summary, KLF4 expression is upregulated in cardiomyocytes treated with Ang II, which subsequently induces the expression of SIAT7A. The elevated levels of SIAT7A, in turn, enhance the transcription of *KLF4*. These findings suggest a positive feedback loop between KLF4 and SIAT7A‐Sialyl‐Tn, ultimately promoting Ang II‐induced cardiac hypertrophy.

## 1 INTRODUCTION

Previous studies have indicated that chronic uncontrolled hypertension is a common cause of pathological myocardial hypertrophy, which often leads to heart failure, arrhythmia, and sudden death.^1^, ^2^, ^3^ Under long‐term stimulation by mechanical or neurohumoral factors, fetal genes in myocardial nuclei are activated, resulting in cardiac structural remodelling.^3^, ^4^ Numerous transgenic and gene knockout animal studies, alongside clinical case reports, have demonstrated that heightened activity of the renin‐angiotensin‐aldosterone system (RAAS), particularly involving angiotensin II (Ang II) and its receptors, significantly contributes to the pathogenesis of cardiac hypertrophy.^5^ It has been shown that RAAS‐targeting therapy somewhat ameliorates cardiac hypertrophy.^6^ Furthermore, the study of targeted molecules or key signalling pathways that are relevant to RAAS‐related pathological myocardial hypertrophy may promote the clinical treatment of cardiac hypertrophy.

Glycosylation is an important kind of post‐translational modification of protein. Abnormal glycosylation has been implicated in the pathogenesis of arrhythmia, cardiac hypertrophy and heart failure,^7^, ^8^, ^9^, ^10^ suggesting that targeting abnormal glycosylation could offer a promising therapeutic approach for managing cardiomyopathy.^11^ The terminal glycosylation process is often modified by sialyltransferase, which is called ‘sialylation’. Researches have shown that sialylation affects cell adhesion, migration and signalling, regulates glycoprotein function and stability.^12^, ^13^, ^14^ It has been reported that the heart is highly glycosylated and especially sialylated. Aberrant sialylation has been associated with pathological conditions such as dilated cardiomyopathy, hypertrophy, heart failure and congenital disorders.^15^, ^16^ Notably, the level of sialic acid detected in hypertrophic myocardium is much higher than that in normal myocardium.^17^, ^18^ Twenty members of the sialyltransferase family have been found.^14^, ^16^ Sialyltransferase 7A (SIAT7A), the focus of this study, is one of the members of CMPNeu5Ac: GalNAc‐R α2, 6‐sialic acid transferases, namely ST6GalNAcI sialic acid glycosyltransferase. SIAT7A catalyses the synthesis of Sialyl‐Tn antigen (Neu5Acα2‐6GalNAc‐O‐Ser/Thr).^14^, ^19^ Researchers have found that the gene encoding SIAT7A is the only gene that synchronously presents increased expression in three different strains of hypertensive rat models.^20^ We previously showed that SIAT7A was upregulated in hypertrophic myocardium and promoted cardiomyocyte hypertrophy via the activation of the HIF‐1α‐TAK1‐NF‐κB pathway.^21^ However, the upstream mechanisms relevant to regulation of SIAT7A in cardiac hypertrophy have not been clarified.

Krüppel‐like factors (KLFs) are a class of transcription factors with three C2H2 zinc finger structures at the C‐terminal. The N‐terminal is a transcription regulatory domain, which can bind a variety of specific proteins to mediate transcriptional regulation.^22^ Eighteen KLFs have been found in the human genome. KLF4 plays a role in cell growth, differentiation and proliferation.^23^, ^24^ KLF4 has also been shown to be involved in the pathogenesis and development of cardiac hypertrophy.^25^, ^26^, ^27^ Our previous study found that KLF4 transactivates *SIAT7A* gene in ischemic myocardium by binding to the *SIAT7A* promoter region (nt‐655 to −636 bp), jointly promoting myocardial cell apoptosis.^28^ However, the potential role of KLF4 in regulating SIAT7A expression in Ang II‐induced hypertrophic cardiomyocytes remains unclear.

It is widely recognized that pathologic left ventricular hypertrophy is a prevalent pathological manifestation of hypertensive heart disease, with the RAAS playing a crucial role in its pathogenesis.^1^, ^25^ This study aims to deepen the underlying mechanism of the interaction between KLF4 and SIAT7A in the progression of Ang II‐induced cardiac hypertrophy, with potential implications for the identification of novel therapeutic targets in clinical settings. Pharmaceutical agents directed towards these specific molecular targets have the potential to intervene with greater precision in the pathogenesis and progression of cardiac hypertrophy, leading to personalized therapeutic strategies and enhanced treatment outcomes.

## 2 MATERIALS AND METHODS

### 2.1 Selection of human specimens

Specimens were obtained from human heart tissue exhibiting cardiac hypertrophy (*n* = 4) or devoid of any heart disease (*n* = 4). All samples were collected and paraffin‐embedded post‐mortem (see details in the Supplementary material online—Data S1). The extraction of human myocardial tissue was conducted, with the informed consent of family members of participants. The protocol was approved by the Institutional Review Board of Dalian Medical University. All procedures adhered to the principles delineated in the Declaration of Helsinki.

### 2.2 Animals and experimental protocols

Adult male Wistar rats, each weighing about 180 g and free of specific pathogens, were sourced from the Animal Medical Center of Dalian Medical University. All experimental protocols were approved by the Animal Research Committee of Dalian Medical University (AEE21109) and adhered to the Guidelines for Animal Research Institutions and the Guidelines for the Care and Use of Laboratory Animals published by the US National Institutes of Health (2011). A total of 24 rats were randomly allocated into four groups: AAV9‐cTNT GFP‐saline rats (*n* = 6), AAV9‐cTNT GFP‐ Ang II rats (*n* = 6), AAV9‐cTNT *Siat7A*‐saline rats (*n* = 6), and AAV9‐cTNT *Siat7A*‐ Ang II rats (*n* = 6). The AAV9 vector, carrying the cardiac troponin T (cTNT GFP) promoter for driving the expression of GFP (AAV9‐ cTNT GFP) and *Siat7A* (AAV9‐ cTNT *Siat7A*), was procured from Hanbio Biotechnology (Shanghai, China). Following anaesthesia of the rats via intraperitoneal injection of pentobarbital (50 mg/kg), the viral solution (1 × 10^12^ vector genomes vg/mL, 200 μL/rat) was slowly injected through the jugular vein.^21^ Osmotic minipumps (Alzet Model 2004; Durect Corporation, Cupertino, CA, USA) prefilled with Ang II (A9525, Aladdin, A107852, Shanghai, China) or saline were employed for subcutaneous administration 2 weeks after AAV9 carrier injection (see details in the Supplementary material online—Data S1).

### 2.3 Echocardiography

Transthoracic echocardiography was performed on the day 29th following Ang II treatment. Each rat was anaesthetised using 3% isoflurane. Two‐dimensional and M‐mode echocardiography was performed utilizing an MS250 transducer (Vevo 770 system; VisualSonics, Toronto, Ontario, Canada). Data were collected and analysed for three consecutive cardiac cycles per measurement. Left ventricular diameter (LVID), left ventricular posterior wall thickness (LVPW) and left ventricular anterior wall thickness (LVAW) during diastolic and systolic periods were measured, and left ventricular ejection fraction (EF%), left ventricular shortening fraction (FS%) and stroke volume (SV) were calculated.

### 2.4 Histopathology

Myocardial tissue was fixed in 10% neutral formalin for 24 h and subsequently embedded in paraffin. Following dehydration, tissue samples were sectioned into 5‐μm slices and dried to obtain tissue sections. Then, haematoxylin–eosin (HE) staining and immunohistochemical staining were conducted. A camera (Leica, Germany) was employed to randomly capture images of three regions, and the cross‐sectional area of the myocardial fibres was quantified using the ImageJ Analyser.

### 2.5 Cell culture and treatments

A human cardiomyocyte‐like cell line (AC16) was procured from the American Type Culture Collection (Manassas, VA, USA). The cells were cultured in DMEM/F12 medium containing 12% fetal bovine serum (FBS) and incubated in a 5% CO_2_ environment at 37°C. Prior to Ang II exposure, AC16 cells were cultured in serum‐free medium for 12 h to enhance drug treatment efficacy. The treatment concentrations of Ang II were 0.1 and 1 μM, and the total treatment time was 24 h.

### 2.6 Transfection

Stable transfection was utilized to generate cells with either knockdown or overexpression of the *SIAT7A* gene, as previously described.^21^ The effective shRNA sequence targeting *SIAT7A* was 5‐GCTACACGATGAAGGGATAAT‐3, and the scrambled shRNA (shNC) sequence was 5‐TTCTCCGAACGTGTCACGT‐3. Puromycin (1 μg/mL) was used to select stable cells. The expression efficiency was assessed using reverse transcription polymerase chain reaction (RT‐PCR) and western blot analysis (see details in the Supplementary material online—Data S1).

*KLF4* knockdown cells were constructed by transient transfection, following the manufacturer's suggested protocol. *KLF4* siRNA (5‐GGACUUUAUUCUCUCCAAUTT‐3) or NC siRNA (5‐ACGUGACACGUUCGGAGAATT‐3) were transfected into cells with siRNA‐Mate. The final concentration of siRNA was 45.5 nM. Samples were collected after 48–72 h. During this period, the medium was periodically replaced. Both lentivirus and siRNA were provided by GenePharma (Shanghai, China).

### 2.7 Immunofluorescence

AC16 cells, including those deficient in *SIAT7A* or *KLF4* were cultured on coverslips with or without Ang II. The cells were fixed with 4% paraformaldehyde at 4°C for 1.5 h, followed by blocking with 1% bovine serum albumin (Beyotime, Shanghai, China) for 2 h. The cells were incubated overnight at 4°C with primary antibodies against α‐actinin (Proteintech, Wuhan, China; 1:100), Sialyl‐Tn (Abcam, Cambridge, UK; 1:70) and KLF4 (ABclonal, Wuhan, China; 1:100). The cells were then incubated with fluorescent secondary antibody conjugated with Alexa Fluor 594 (Proteintech, 1:100) at room temperature for 1 h. After washing with PBS, the nuclei were counterstained with DAPI (Beyotime, 1:1000) for 15 min. The coverslips were then mounted using an anti‐fade solution (Beyotime). Fluorescence images were captured using a Leica fluorescence microscope, and the cell surface area was measured using ImageJ Analyser, with the average calculated.

### 2.8 RNA extraction and quantitative real‐time RT‐PCR (qRT‐PCR) assay

Total RNA was extracted using Trizol (Ambion, Shanghai, China). Total RNA was reverse‐transcribed into cDNA using a reverse transcription kit (Vazyme, Nanjing, China). Primers were provided by Jin kairui (Wuhan, China). qRT‐PCR was conducted utilizing ChamQ Universal SYBR Qpcr Master Mix (Vazyme, Nanjing, China).

### 2.9 Western blot analysis

Total protein was extracted from myocardial tissue or AC16 cell lysates. The protein concentration was determined using the bicinchoninic acid (BCA) method. Equal amounts of protein samples were then separated by 10% sodium dodecyl sulfate‐polyacrylamide gel electrophoresis (SDS‐PAGE) and subsequently transferred onto a PVDF membrane. The membrane was blocked with 5% skim milk for 1 h at room temperature, and incubated with primary antibodies against GAPDH (Proteintech, 1:5000), ANP (Proteintech, 1:800), α‐actinin (Proteintech, 1:1000), KLF4 (Proteintech, 1:1000) and SIAT7A (Abcam, 1:500) at 4°C overnight. Following this, the membranes were incubated with a secondary antibody conjugated with horse radish peroxidase for 1 h at room temperature. Protein bands were then detected using an enhanced chemiluminescence liquid kit (Yazyme) and visualized with a chemiluminescence gel imaging system. Image Lab analysis software was used to analyse the strip images.

### 2.10 Luciferase assay

To generate the *KLF4* luciferase reporter, a 2000‐bp promoter fragment of the human *KLF4* gene was amplified via PCR and cloned into the pGL3 plasmid. All constructs were confirmed by DNA sequencing. The primers used for PCR amplification are detailed in the Supplementary Information—Data S1. AC16 cells, either with stable knockdown or overexpression of *SIAT7A*, as well as scramble‐transfected AC16 cells, were seeded in 96‐well plates. After 24 h, the *KLF4* luciferase reporters and Renilla plasmid were co‐transfected for an additional 24 h. Luciferase activities were then measured using a dual luciferase reporter assay detection kit (Promega Corporation, USA).

### 2.11 Statistical analysis

Results were expressed as mean ± standard error of the mean, derived from at least three independent experiments. For comparisons between two groups, the *t*‐test was employed, ensuring data met criteria for independence, normality, and variance homogeneity. For comparisons involving multiple groups, ANOVA was conducted, followed by a post‐hoc Tukey test. Statistical significance was determined at a *p* <  0.05. All statistical analyses were carried out using SPSS software, version 22.0 (SPSS Inc.).

## 3 RESULTS

### 3.1 KLF4 and SIAT7A are increased in hypertrophic myocardium of essential hypertension and in hypertrophic cardiomyocytes induced by Ang II

**FIGURE 1:**
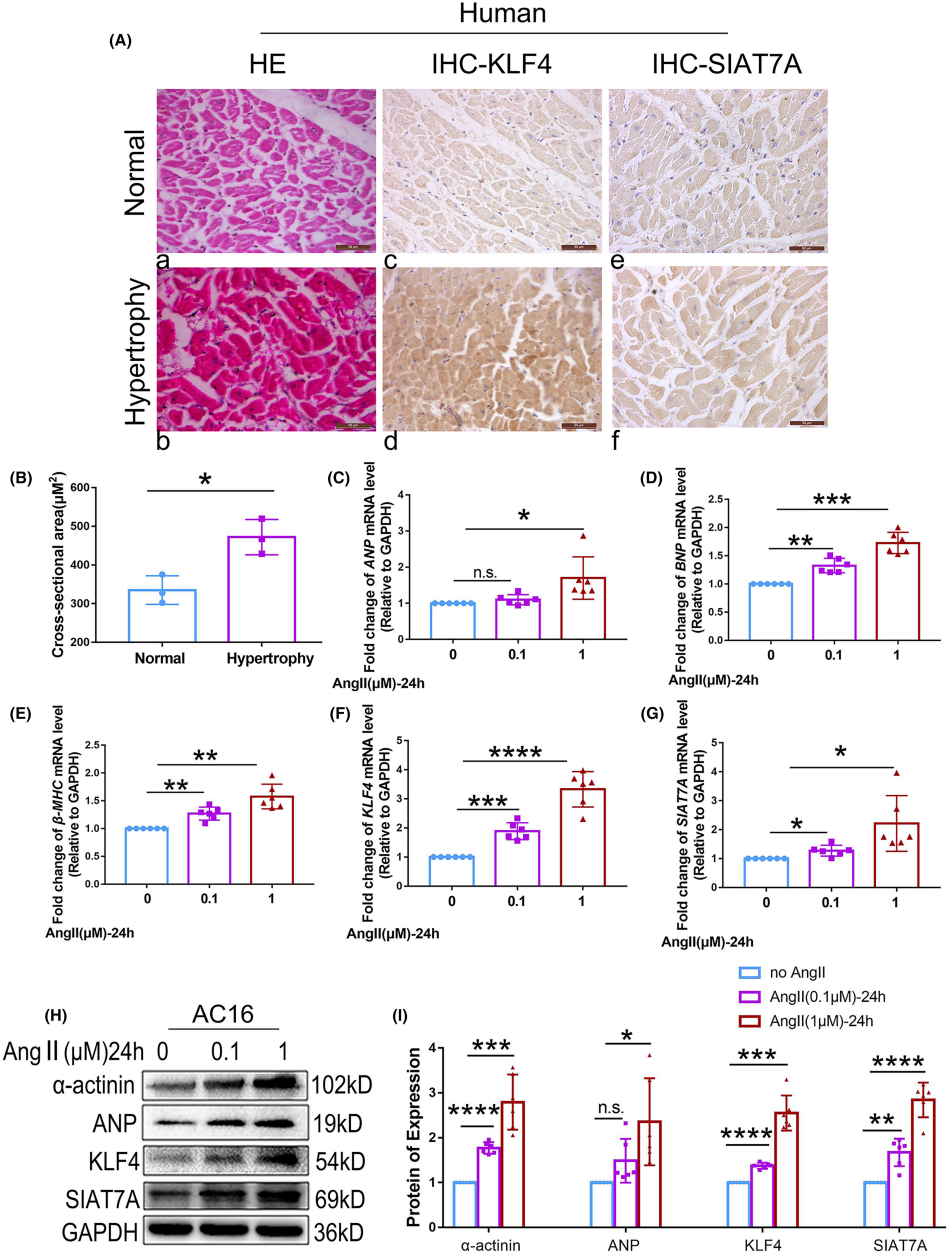
Expression of KLF4 and SIAT7A in hypertensive hypertrophy of the myocardium and hypertrophic cardiomyocytes. (A) Representative HE and IHC images of human left ventricles. The morphology of normal and hypertrophic human myocardium is shown (a and b). Positive immunoreactive signals for KLF4 and SIAT7A expression are shown as brown deposits in the cell (c–f). (B) Assessment of the myocyte cross‐sectional area (*n* = 3/group). AC16 cells were stimulated with 0, 0.1 and 1 μM Ang II for 24 h. mRNA expression level of (C) *ANP*, (D) *BNP*, (E) *β‐MHC*, (F) *KLF4* or (G) *SIAT7A* was detected. (H, I) Representative immunoblots and statistical analysis of protein expression of α‐actinin, ANP, KLF4, and SIAT7A. The results are shown as the mean ± SD of six independent experiments. n.s., not significant; *p < 0.05; **p < 0.01; ***p < 0.001; ****p < 0.0001.

HE staining was performed to identify myocardial hypertrophy in human myocardial tissue. Immunohistochemical staining was used to detect the expression of KLF4 and SIAT7A in hypertrophic myocardium (Figure 1A). Compared with normal myocardium (Figure 1A‐a), larger cross‐sectional area and narrowed cell gap were obviously visible in human hypertrophic myocardial fibres (Figure 1–A‐b,B). The expression of KLF4 and SIAT7A were stronger in the cytoplasm of hypertrophic cardiac myocytes than that in the cytoplasm of normal cardiac myocytes (Figure 1A‐c to f).

In vitro, the effects of Ang II on the expression of KLF4 and SIAT7A were examined in cultured human cardiomyocyte cells (AC16) at both the RNA and protein levels. As shown in Figure 1C,E,H,I, the levels of ANP, BNP, β‐MHC and α‐actinin increased significantly in AC16 cells treated with Ang II in a concentration‐dependent manner. This indicates that Ang II stimulation resulted in cardiomyocyte hypertrophy. Additionally, the expression levels of KLF4 and SIAT7A were also upregulated in a concentration‐dependent manner in AC16 cells treated with Ang II (Figure 1F,G,H‐a,I).

### 3.2 *KLF4* knockdown decreases expression of SIAT7A and Sialyl‐Tn, and attenuates cardiomyocyte hypertrophy

**FIGURE 2:**
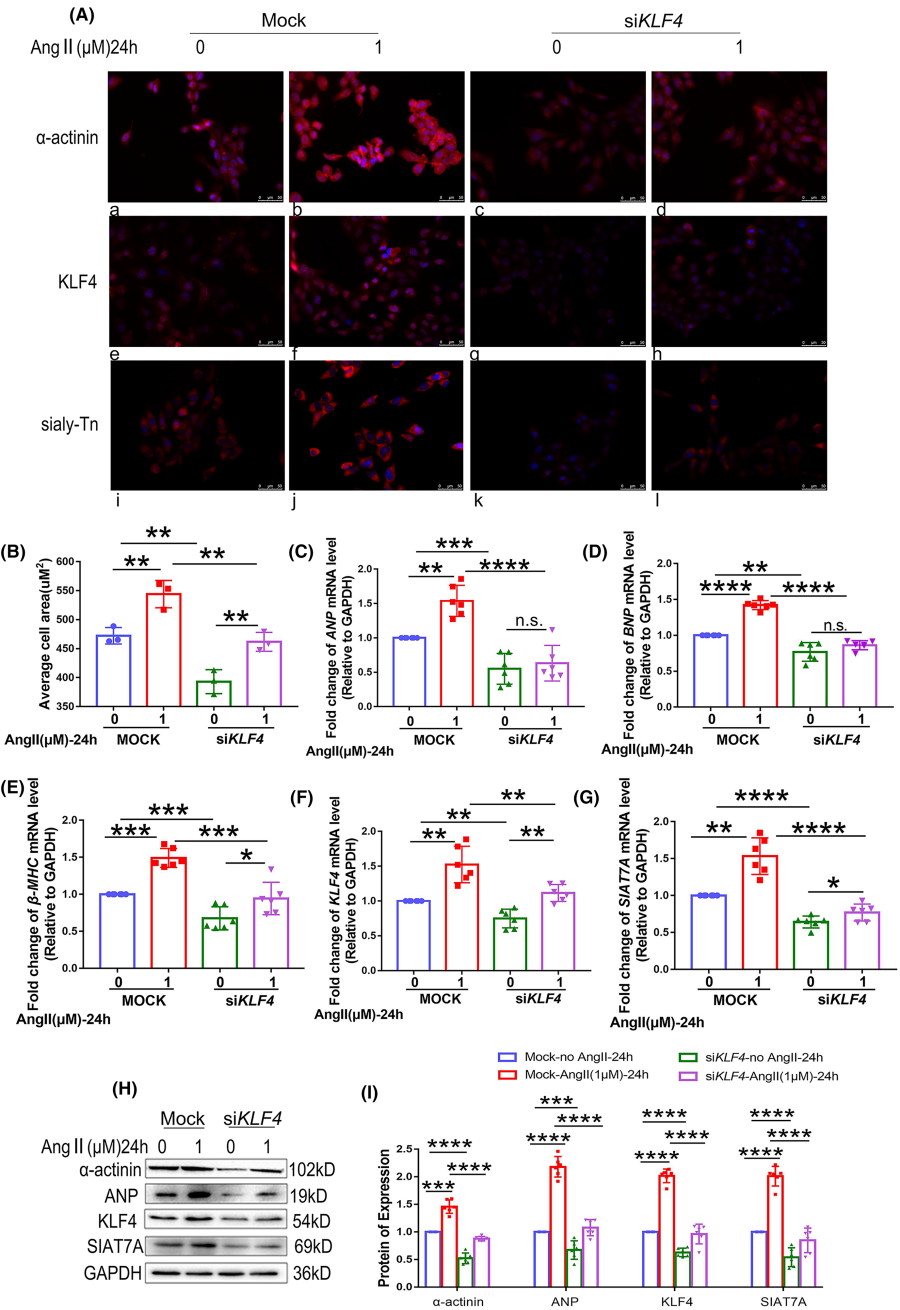
Effects of *KLF4* knockdown on expression of SIAT7A and Sialyl‐Tn in hypertrophic cardiomyocytes. AC16 cells with *KLF4* knockdown were treated with saline water or 1 μM Ang II for 24 h. (A) Representative images of immunofluorescence staining for α‐actinin, KLF4, and Sialyl‐Tn in mock or si*KLF4* AC16 cells. Red signals were positive immunoreactions. Blue signals were nuclei counterstained with DAPI. The upper panels display a‐actinin expression (a–d). The middle panels display KLF4 expression (e–h). The lower panels display Sialyl‐Tn expression (i–l). (B) Assessment of the average area of AC16 cells (*n* = 3/group). (C) *ANP*, (D) *BNP*, (E) *β‐MHC*, (F) *KLF4*, or (G) *SIAT7A* mRNA expression was examined. (H, I) Representative immunoblots and statistical analysis of protein expression of α‐actinin, ANP, KLF4 and SIAT7A. The results are shown as the mean ± SD of six independent experiments. *p < 0.05; **p < 0.01; ***p < 0.001; ****p < 0.0001.

In vitro, the effects of *KLF4* knockdown on the expression of SIAT7A and Sialyl‐Tn were checked in cultured AC16 cells at both the RNA and protein levels and by morphological analyses. As shown in Figure 2A(a–d),B–E,H,I the average cell area and the levels of ANP, BNP, β‐MHC and α‐actinin were significantly enhanced in AC16 cells transfected by shRNA NC after treatment with Ang II. The reduction of *KLF4* expression resulted in a notable decrease in cell area and the elevated levels of these markers induced by Ang II in AC16 cells. This suggests that *KLF4* knockdown can inhibit cardiac myocyte hypertrophy. Additionally, significantly decreased expression levels of SIAT7A and Sialyl‐Tn were detected in AC16 cells transfected with si*KLF4* (Figure 2A,G,I, and Figure S1).

### 3.3 *SIAT7A* knockdown decreases expression of KLF4 and Sialyl‐Tn and attenuates cardiomyocyte hypertrophy

**FIGURE 3:**
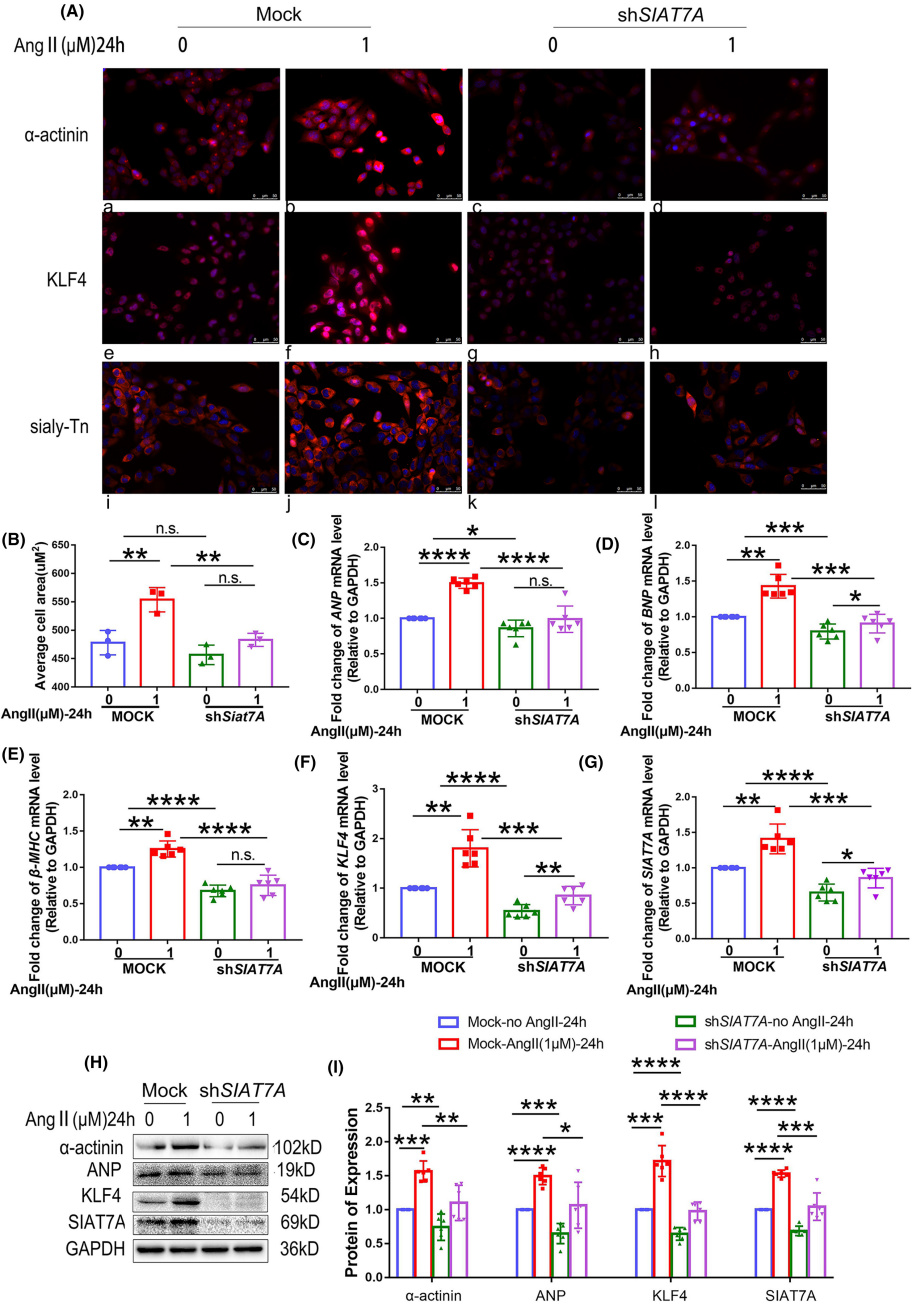
Effects of *SIAT7A* knockdown on expression of KLF4 and Sialyl‐Tn in hypertrophic cardiomyocytes. AC16 cells with *SIAT7A* knockdown (sh*SIAT7A* AC16 cells) were treated with saline water or 1 μM Ang II for 24 h. (A) Representative images of immunofluorescence staining for α‐actinin, KLF4 and Sialyl‐Tn in mock or sh*SIAT7A* AC16 cells. Red signals were positive immunoreactions. Blue signals were nuclei counterstained with DAPI. Upper panels display α‐actinin expression (a–d). Middle panels display KLF4 expression (e–h). Lower panels display Sialyl‐Tn expression (i–l). (B) Assessment of the average area of AC16 cells and sh*SIAT7A* AC16 cells (n = 3/group). (C) *ANP*, (D) *BNP*, (E) *β‐MHC*, (F) *KLF4* or (G) *SIAT7A* mRNA expression was examined. (H, I) Representative immunoblots and statistical analysis of protein expression of α‐actinin, ANP, KLF4, and SIAT7A. Results are shown as the mean ± SD of six independent experiments. *p < 0.05; **p < 0.01; ***p < 0.001; ****p < 0.0001.

In vitro, shRNA *SIAT7A* was transferred into cultured AC16 cells, and the effects of *SIAT7A* knockdown on the expression of KLF4 and Sialyl‐Tn were examined at both RNA and protein levels and by morphological analyses. Downregulation of *SIAT7A* significantly decreased the average cell area and the high levels of ANP, BNP, β‐MHC and α‐actinin in AC16 cells treated with Ang II (Figure 3A–E,H–I). This indicates that cardiomyocyte hypertrophy was inhibited by *SIAT7A* knockdown. Transfection with shRNA *SIAT7A*, as opposed to shRNA NC, significantly reduced the expression of KLF4 at both the RNA and protein levels, as well as the expression of Sialyl‐Tn in the morphology of AC16 cells, regardless of Ang II treatment (Figure 3A,F,H–I and Figure S2). This shows that *SIAT7A* knockdown restricted expression of KLF4 and Sialyl‐Tn in both normal and hypertrophic cardiomyocytes.

### 3.4 *SIAT7A* overexpression increases expression of KLF4 and promotes cardiomyocyte hypertrophy

**FIGURE 4:**
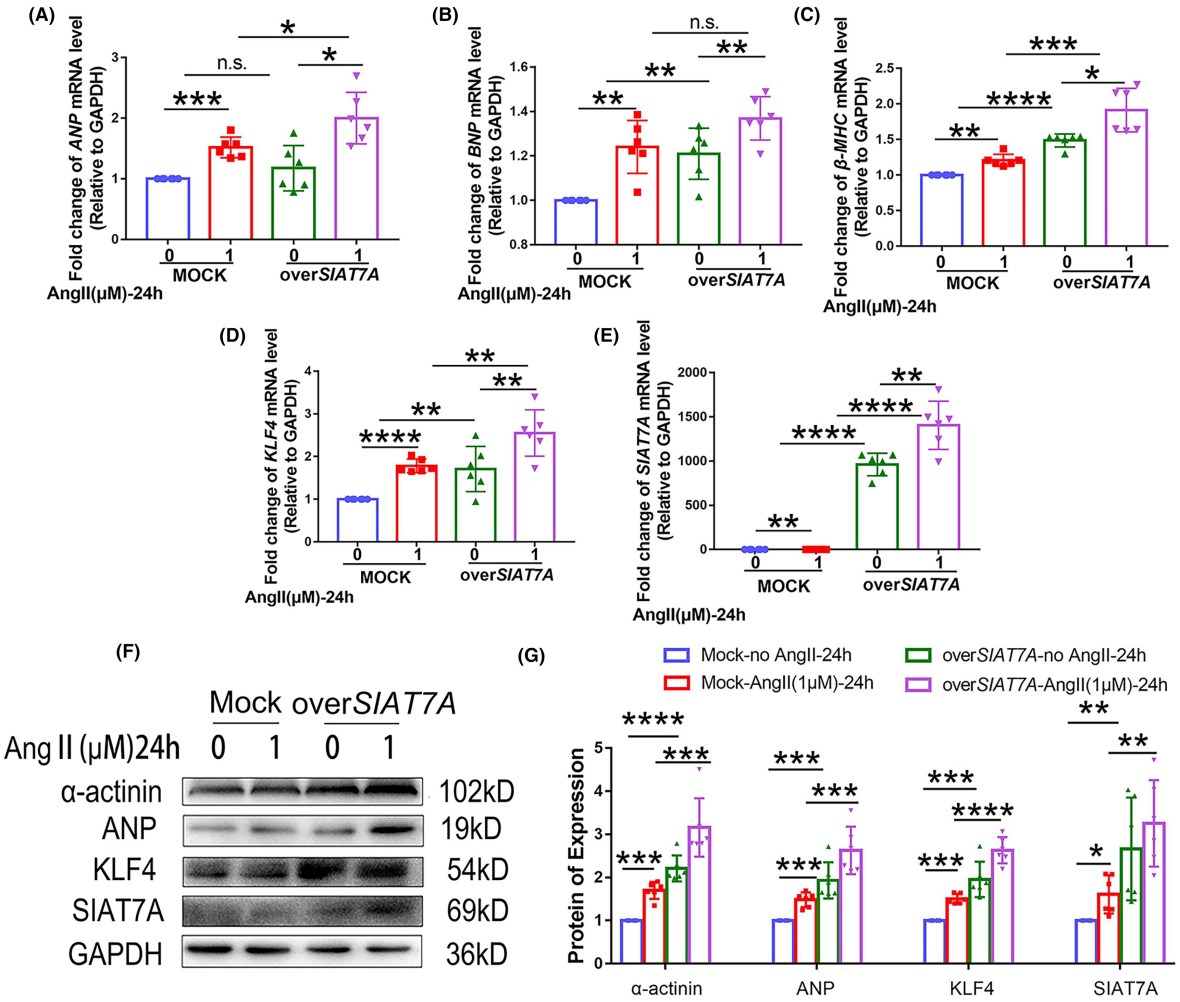
Effects of *SIAT7A* overexpression on expression of KLF4 in hypertrophic cardiomyocytes. AC16 cells overexpressing *SIAT7A* were treated with saline water or 1 μM Ang II for 24 h. (A) *ANP*, (B) *BNP*, (C) *β‐MHC*, (D) *KLF4* or (E) *SIAT7A* mRNA expression was examined. (F, G) Representative immunoblots and statistical analysis of protein expression of α‐actinin, ANP, KLF4 and SIAT7A. The results are shown as the mean ± SD of six independent experiments. n.s., not significant; *p < 0.05; **p < 0.01; ***p < 0.001; ****p < 0.0001.

In vitro, *SIAT7A* was overexpressed in cultured AC16 cells. Compared with the mock cells, overexpression of *SIAT7A* significantly enhanced the levels of ANP, BNP, β‐MHC and α‐actinin (Figure 4A–C,F,G), and increased the expression of KLF4 in the AC16 cells treated or not treated with Ang II (Figure 4D,F,G).

**FIGURE 5:**
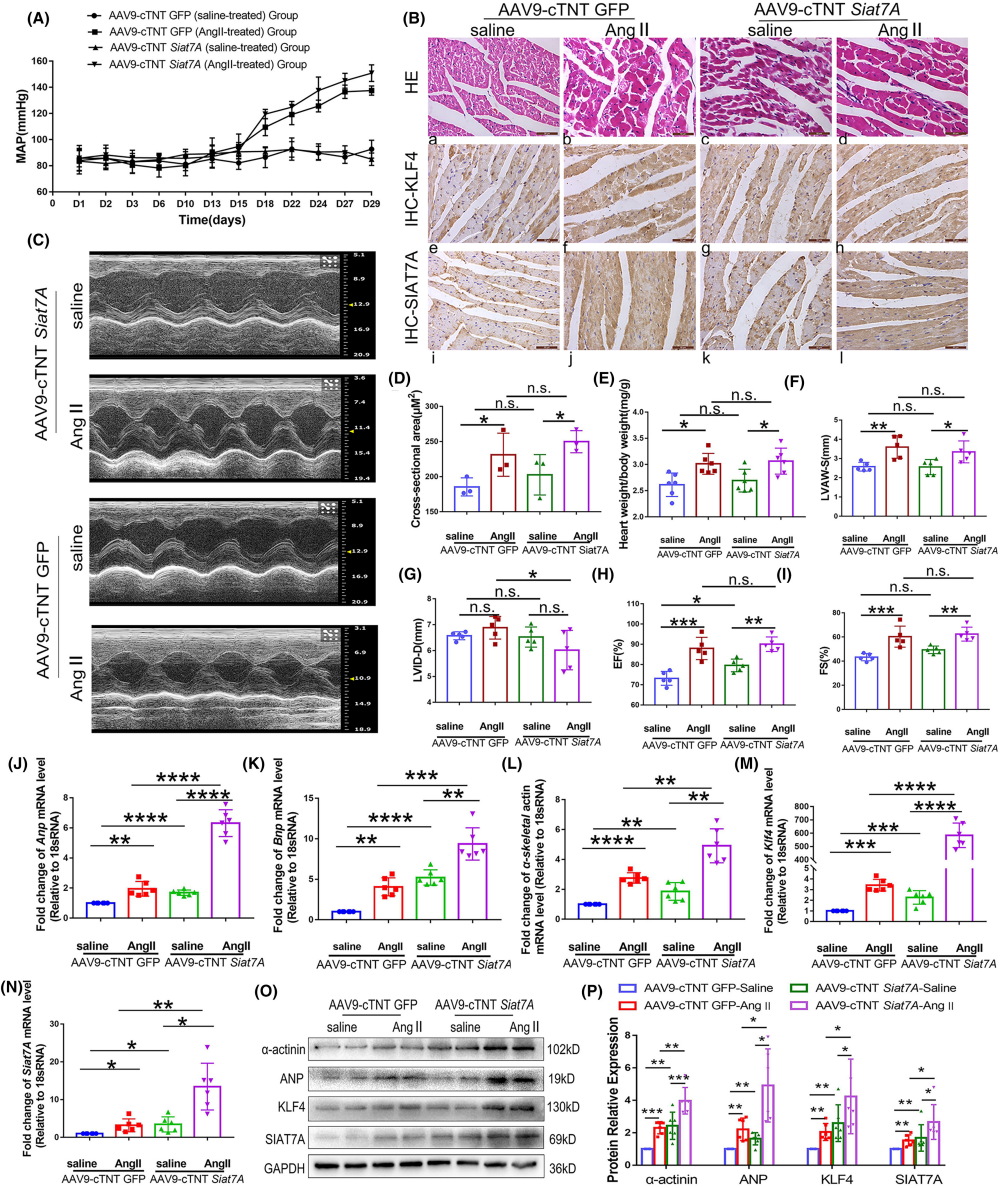
Effect of *Siat7A* overexpression on the expression of KLF4 and cardiac hypertrophy in vivo. (A) Recordings for MAP (*n* = 6/group). (B) Representative HE and IHC images of rat left ventricular wall. The upper panels display HE staining showing the morphology of cardiomyocytes. The lower panels display the IHC images of KLF4 and SIAT7A expression. Positive immunoreactive signals for KLF4 and SIAT7A expression are shown by brown deposits in the cell. (C) Representative M‐mode echocardiography of left ventricular chambers. Assessment of left ventricular LVAW‐s (F), LVID‐d (G), EF% (H) and FS% (I) (*n* = 5/group). (D) Assessment of the myocyte cross‐sectional area of rats. (E) Quantification results of HW/BW (*n* = 6/group). The analysis of mRNA level of *ANP* (J), *BNP* (K), *α‐actin* (L), *KLF4* (M), and *SIAT7A* (N) in the left ventricular myocardium of rats (*n* = 6/group). (O, P) Representative immunoblots and statistical analysis of the expression levels of α‐actinin, ANP, KLF4, and SIAT7A in the ventricular myocardium of rats (*n* = 6/group). The results are shown as the mean ± SD. n.s., not significant; *p < 0.05; **p < 0.01; ***p < 0.001; ****p < 0.0001.

In vivo, the changes in cardiac structure remodelling and contractile function, as well as expression of KLF4 and SIAT7A, were examined in rats treated by Ang II. As shown in Figure 5, Ang II treatment elevated mean arterial pressure (MAP, Figure 5A) and induced cardiac hypertrophic manifested by increased cardiomyocyte size (Figure 5A,B‐a to d,D), HW/BW ratio (Figure 5E), LVAW‐S (Figure 5F) and reactivations of myocardial fetal genes ANP, BNP and α‐actinin (Figure 5J–L,O–P). Additionally, EF% (Figure 5H) and FS% (Figure 5I) of the LV were increased. Consistent with the in vitro results, continuous infusion of Ang II significantly increased the expression of SIAT7A and KLF4 at both protein and mRNA levels in the rat myocardium (Figure 5B,M–P).

To assess the impact of *Siat7A* overexpression on cardiac structure remodelling, contractile function, and KLF4 expression under pathological stress in vivo, an AAV9 vector encoding *Siat7A* under the control of the cardiac‐specific cTNT promoter was administered to rats, followed by a continuous infusion of Ang II for 4 weeks. Compared to AAV9‐cTNT GFP rats, *Siat7A* overexpression led to a further increase in KLF4 expression (Figure 5B,M,O–P), more severe cardiac hypertrophic responses manifested by further reactivations of myocardial fetal genes (Figure 5J–L,O–P). The in vivo and in vitro findings indicate that the overexpression of *Siat7A* leads to an upregulation of KLF4 levels in cardiomyocytes, thereby promoting cardiac hypertrophy.

### 3.5 KLF4 expression precedes that of SIAT7A during the progression of cardiomyocyte hypertrophy

**FIGURE 6:**
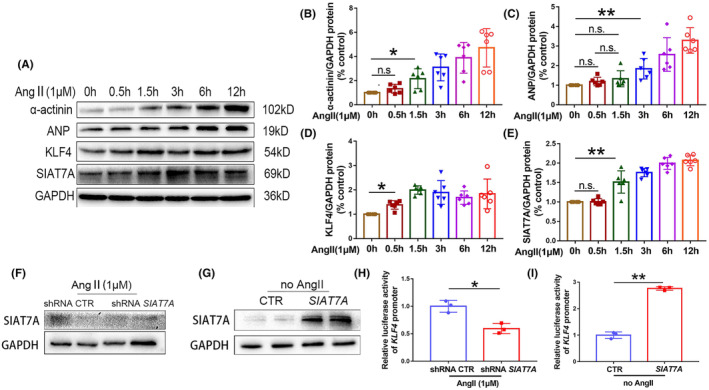
The interaction between KLF4 and SIAT7A during the development of myocardial hypertrophy. AC16 cells were treated with sterile water or 1 μM Ang II for 0.5, 1.5, 3, 6, or 12 h. (A) Representative immunoblots for expression of α‐actinin, ANP, KLF4, and SIAT7A. Protein expression of (B) α‐actinin, (C) ANP, (D) KLF4 and (E) SIAT7A was analysed. The results are shown as the mean ± SD of six independent experiments. The transcription activity of *KLF4* promoter was determined by luciferase reporter assays. The *KLF4* luciferase reporters were transiently transfected into AC16 cells that were either stably depleted or stably overexpressing *SIAT7A*, followed by Ang II stimulation. Representative immunoblots illustrating SIAT7A expression are presented in panels (F) and (G). The transcriptional activity was subsequently assessed, as depicted in panels (H) and (I). The results are shown as the mean ± SD of three independent experiments. n.s., not significant; *p < 0.05; **p < 0.01.

To clarify the positive feedback between KLF4 and SIAT7A in hypertrophic cardiomyocytes, the AC16 cells were treated with Ang II for 0.5, 1.5, 3, 6, and 12 h, and then the expression levels of KLF4, SIAT7A and hypertrophic markers were detected. As shown in Figure 6A,D–E, increased expression of KLF4 and SIAT7A in the AC16 cells were detected at 0.5 and 1.5 h, respectively. Cardiac myocytes began to show signs of hypertrophy at 1.5 h, given the increased expression of α‐actinin (Figure 6A,B). These results suggested that KLF4 was initially expressed, and subsequently initiated SIAT7A expression during the development of cardiac hypertrophy induced by Ang II.

### 3.6 SIAT7A upregulates the transcriptional regulation of *KLF4* promoter in cardiomyocytes

Given that SIAT7A was found to mediate KLF4 expression, a luciferase assay was conducted to investigate its effect on the transcription activity of *KLF4* promoter. We verified that the expression of SIAT7A was downregulated by shRNA *SIAT7A* transfection and upregulated by overexpression of the *SIAT7A* gene in AC16 cells (Figure 6F,G). Correspondingly, depletion of *SIAT7A* significantly decreased the activity of *KLF4* reporter (Figure 6H), while overexpression of *SIAT7A* significantly increased it (Figure 6I). These results suggest that SIAT7A‐mediated upregulation of *KLF4* expression on RNA level depends on increased transcription activity of *KLF4* promoter.

**FIGURE 7:**
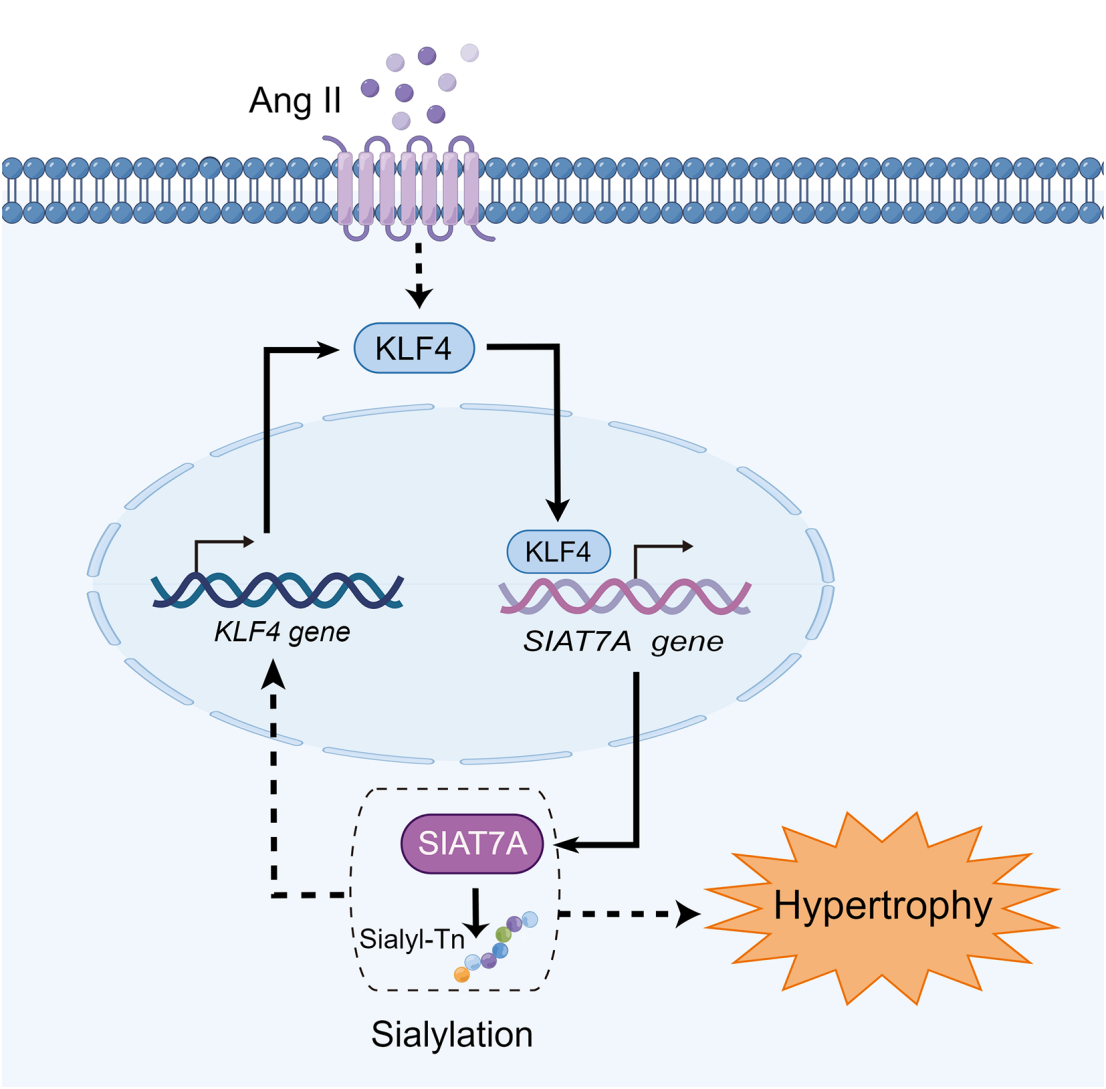
Schematic representation of the positive feedback loop between KLF4 and SIAT7A‐Sialyl‐Tn that promotes cardiac hypertrophy following Ang II stimulation. KLF4 expression is upregulated in cardiomyocytes treated by Ang II, which initiates SIAT7A and Sialyl‐Tn expression. The increased SIAT7A in turn promotes the transcription of *KLF4*. Thereafter, a positive feedback loop between KLF4 and the SIAT7A‐Sialyl‐Tn is formed that promotes cardiac hypertrophy induced by Ang II. The solid line represents direct action, while the dashed line represents indirect action, which needs further study.

## 4 DISCUSSION

In this study, we observed a concurrent increase in SIAT7A and KLF4 levels in the hypertrophic myocardium of patients with essential hypertension, rats and Ang II‐induced hypertrophic AC16 cells. Knockdown of *KLF4* reduced the expression level of SIAT7A and Sialyl‐Tn, thereby inhibiting cardiomyocyte hypertrophy induced by Ang II. Knockdown of *SIAT7A* decreased the expression of KLF4 by downregulating the transcription activity of *KLF4* promoter and inhibited cardiomyocyte hypertrophy. Moreover, overexpression of *SIAT7A* promoted the expression of KLF4 by upregulating the transcription activity of *KLF4* promoter and aggravated cardiomyocyte hypertrophy. In addition, KLF4 may be initially activated and subsequently trigger the expression of SIAT7A in the cardiac hypertrophy process induced by Ang II.

KLF4 is a zinc finger‐type transcription factor expressed in a various tissues, including the cardiovascular system, and is involved in multiple physiological and pathological processes.^23^ Elevated KLF4 expression has been observed in neonatal rat ventricular myocytes (NRVM) treated with Ang II or phenylephrine (PE), and in the hypertrophic hearts of mice receiving transverse aorta constriction (TAC) or a continuous infusion of isoproterenol (ISO) or Ang II.^29^, ^30^, ^31^ In our previous study, we demonstrated that KLF4 directly binds to the *SIAT7A* promoter (nt‐655 to −636 bp) and upregulates SIAT7A expression in ischemic myocardium, thereby contributing to cardiomyocyte apoptosis.^28^ Furthermore, SIAT7A has been shown to promote cardiomyocyte hypertrophy.^21^ To determine how KLF4 expression changes and whether it is related to SIAT7A in cardiomyocyte hypertrophy, we detected the expression of KLF4 and SIAT7A at both the translational and transcriptional level in a human cardiomyocyte cell line treated with different concentrations of Ang II. We found that KLF4 expression was elevated by Ang II stimulation, concomitant with increased expression of SIAT7A and induction of cardiac hypertrophy. Moreover, *KLF4* knockdown decreased expression of SIAT7A and Sialyl‐Tn induced by Ang II stimulation, thereby inhibiting cardiac hypertrophy. These findings suggest that KLF4 may mediate Ang II‐induced cardiomyocyte hypertrophy via the upregulated transcription and translation of *SIAT7A* and the synthesis of Sialyl‐Tn. Moreover, concurrent increased levels of KLF4 and SIAT7A were also noted in the left ventricular myocardium of hypertensive patients and in the hypertensive hypertrophic myocardium induced by Ang II in rats. Pathologic left ventricular hypertrophy is recognized as the prevalent pathological alteration in hypertensive heart disease, with the RAAS implicated in its genesis and progression.^3^, ^32^, ^33^ Thus, our results indicate that the upregulation of KLF4 induced by Ang II can promote the expression of SIAT7A, thereby promoting hypertensive cardiac hypertrophy.

Our study revealed another intriguing phenomenon: the expression levels of KLF4 and SIAT7A mutually influence each other's transcription and translation. Given that KLF4 is a transcription factor of *SIAT7A* in the ischemic myocardium^28^; it follows that *KLF4* knockdown decreased the expression of SIAT7A and Sialyl‐Tn in hypertrophic cardiomyocytes treated by Ang II. Conversely, *SIAT7A* knockdown, decreased the expression levels of KLF4 and Sialyl‐Tn, while when *SIAT7A* overexpression, both in vivo and in vitro, increased the expression level of KLF4. These findings underscore a dynamic interplay between KLF4 and SIAT7A‐Sialyl‐Tn during the development of pathological myocardial hypertrophy, indicating a complex regulatory network between these genes with potential implications for a deeper understanding of gene interactions.

To detect the mechanism of the interaction between KLF4 and SIAT7A, a time‐series experiment and a luciferase reporter assay were conducted. When cardiomyocytes were stimulated with Ang II for different time points, the expression levels of KLF4 and SIAT7A, as well as those of hypertrophy markers, were found to change in a time‐dependent manner. Notably, KLF4 expression increased prior to SIAT7A, suggesting KLF4 initiates *SIAT7A* activity during Ang II‐induced cardiac hypertrophy. Furthermore, the luciferase reporter assay verified that SIAT7A‐ Sialyl‐Tn exerted positive feedback on KLF4 expression via upregulating the transcription activity of *KLF4* promoter. SIAT7A, a glycosyltransferase, catalyses the synthesis of Sialyl‐Tn antigen, which is associated with various glycoproteins, including transcription factors.^34^ It is well‐established that glycosylation can regulate the transcriptional activity of promoters through diverse modifications in transcription factor properties, including localization, stability and transcriptional activation.^34^, ^35^ And it has also been reported that glycosylation increases the stability of transcription factors that transactivate fetal genes during cardiac hypertrophy.^36^, ^37^ Therefore, we hypothesize that SIAT7A, acting as a glycosyltransferase, enhances *KLF4'*s transcriptional activity by sialylation of specific *KLF4* transcription factors. Our future research will focus on elucidating the precise mechanisms by which SIAT7A regulates KLF4.

In conclusion, our present study identified an interaction between KLF4 and SIAT7A‐Sialyl‐Tn during the development of myocardial hypertrophy. KLF4 expression is upregulated in cardiomyocytes treated by Ang II, which subsequently initiated SIAT7A expression. This interaction establishes a positive feedback loop between KLF4 and the SIAT7A‐Sialyl‐Tn, there by promoting Ang II induced cardiac hypertrophy (Figure 7). Investigating the molecular interactions between these two entities holds potential for developing innovative precision therapeutic strategies targeting hypertensive myocardial hypertrophy. Nevertheless, translating these findings into practical clinical interventions requires further validation of their efficacy and safety through rigorous clinical trials.

## AUTHOR CONTRIBUTIONS

**Qiying Yao:** Data curation (equal); visualization (lead); writing – original draft (lead); writing – review and editing (lead). **Xinrui Hu:** Data curation (lead); formal analysis (lead); investigation (equal). **Tiantian Bian:** Investigation (equal). **Qing Zhang:** Validation (equal). **Zhao Xue:** Investigation (equal). **Yuesheng Lv:** Methodology (equal). **Shupeng Ren:** Validation (equal). **Yue Chen:** Investigation (equal). **Dongmei Zhang:** Conceptualization (lead); project administration (equal); resources (lead); writing – review and editing (supporting). **Liang Chen:** Funding acquisition (lead); project administration (equal); supervision (equal).

## CONFLICT OF INTEREST STATEMENT

The authors declare that there are no conflicts of interest.

## Supporting information


**Data S1:** Supporting Information.

## Data Availability

The data that support the findings of this study are available from the corresponding author upon reasonable request.
