# Supplementary material for: Role of KLF4 and SIAT7A interaction accelerates myocardial hypertrophy induced by Ang II
Source: J Cell Mol Med. 2024 Oct 21;28(20):e70144. doi: 10.1111/jcmm.70144 (PMC11492152; doi:10.1111/jcmm.70144)
Supplement: Supplementary file 1 — Data S1: Supporting Information. [file JCMM-28-e70144-s001.docx]

**Detailed Methods**

**2. Materials and Methods**

**2.1 Selection of human specimens**

The four human heart tissues exhibiting cardiac hypertrophy were sourced from patients who had succumbed to myocardial infarction. These patients had a documented history of hypertension, were exclusively male, aged between 50 and 60 years, and demonstrated a left ventricular anterior wall thickness exceeding 1.5cm.

 In contrast, the four human heart tissues without cardiac hypertrophy were procured from autopsy specimens of individuals who had died due to traffic accidents, brain aneurysm ruptures, or brainstem hemorrhages. None of these individuals had a history of hypertension, and their ages ranged from 30 to 50 years.

Extraction of human myocardial tissue was conducted following the acquisition of informed consent from family members of participants. The protocol received approval from the Institutional Review Board of Dalian Medical University (Approval No.2021-002). All procedures adhered to the ethical principles delineated in the Declaration of Helsinki.

**2.2 Animals and experimental protocols**

**2.2.1 Administration of angiotensin II utilizing osmotic pump in vivo**

The osmotic minipumps (Alzet Model 2004; Durect Corporation, Cupertino, CA, USA) employed in our study are engineered to deliver a sustained release of 200μL of solution over a four-week period. These minipumps were conditioned in saline at 37 ℃ overnight to ensure optimal functioning. Taking into account the weight of the rats and the penetration rate of the osmotic minipumps, which is 200 ng/kg/min, we accurately determined the necessary volumes of 0.01% acetic acid and Ang II reserve solution (50 mg/mL) for each rat. The two solutions were then meticulously combined, and 200 μL of the resulting mixture was loaded into each osmotic minipumps.

Following the priming procedure, the minipump was surgically implanted subcutaneously via a small incision (approximately 1.5 cm in length) made in the rostral midscapular region of the anesthetized rat. Ang II was continuously infused at a rate of 200 ng/kg/min over the four-week experimental period.

Blood pressure measurements were obtained daily using the tail-cuff system (BP-98A, Softron, Japan). For each rat, the blood pressure values from four consecutive measurement cycles were averaged to ensure data accuracy and consistency.

**2.2.2 Sacrifice of rats**

For the animal model, rats were subjected to a 12-hour fasting period and subsequently anesthetized via intraperitoneal injection of sodium pentobarbital. Upon achieving adequate anesthesia, the hearts were exposed and harvested for subsequent experimental analysis.

**2.3 Cellular experimental protocols**

**2.3.1 Cell culture and Angiotensin II Treatment**

A human cardiomyocyte-like cell line (AC16) was procured from the American Type Culture Collection (Manassas, VA, USA) and maintained in DMEM/F12 cell medium supplemented with 12% fetal bovine serum (FBS). The cells were incubated under standard conditions of 5% CO_2_ at 37°C.

Upon reaching 40-50% confluence, the medium was replaced with serum-free medium to induce cellular starvation for a duration of approximately 12 hours. Following this starvation period, 4mL of Ang II at various concentrations (0 μM, 0.1 μM, 1 μM) diluted in serum-free medium was administered, with the medium being refreshed every 6 hours. After a 24-hour exposure to Ang II, samples were collected for subsequent analysis.

**2.3.2 Stable Transfection**

To establish a stable cell line, 100 μL of lentivirus solution (LV5-Siat7A, 3x10^8^ TU/mL; LV5-NC, 1x10^8^ TU/mL; GenePharma, China) and 8μL of polybrene (5 μg/mL) were added to 8mL of medium and gently mixed before being added to AC16 cells. After 6 hours, the medium was replaced with a medium containing puromycin (1 μg/mL) to select for stably expressing cell lines. Microscopic observation indicated that the transfection efficiency exceeded 90%, allowing for subsequent experiments. The expression efficiency was evaluated using Western blot analysis and real-time RT-PCR analysis.

**2.3.3 Transient Transfection**

Small interfering *KLF4* sequences were synthesized by GenePharma Co., Ltd. (5'-GGACUUUAUUCUCUCCAAUTT-3') or NC siRNA (5'-ACGUGACACGUUCGGAGAATT-3'). Transient transfection was performed when the cell confluence in the six-well plate reached 40-50%. The siRNA transfection complex was prepared by combining 125 μL of serum-free medium with 5 μL of siRNA oligo (20 μM) / Lipofectamine 3000. The mixture was thoroughly stirred and evenly distributed into the petri dish, followed by incubation at 37 ℃ with 5% CO_2_ for 12 hours. Subsequently, the medium in the six-well plate was replaced with serum-free medium for an additional 12 hours of starvation. Different concentrations of angiotensin II prepared in a serum-free medium were then administered. Protein samples were harvested 24 hours post-treatment, while RNA samples were collected 48 hours post-treatment for subsequent experiments.

**Table S1. Antibody used for Immunofluorescence and Western blot**

| Antibody | Code | Company |
| --- | --- | --- |
| ST6GalNAcⅠantibody | ab229816 | Abcam |
| KLF4 Polyclonal antibody | 11880-1-AP | Proteintech |
| Sialyl-Tn antibody | ab76756 | Abcam |
| NPPA rabbit polyclonal antibody | 27426-1-AP | Proteintech |
| Alpha actinin rabbit polyclonal antibody | 11313-2-AP | Proteintech |
| GAPDH mouse monoclonal antibody | 60004-1-lg | Proteintech |
| HRP-conjugatedAffinipure GoatAnti-RabbitIgG（H+L） | SA00001-2 | Proteintech |
| HRP-conjugatedAffinipure GoatAnti-MouseIgG（H+L） | SA00001-1 | Proteintech |

**Table S2. Primers used for Real-time PCR**

| Gene | Primer sequence |
| --- | --- |
| Human-*SIAT7A* | F:5-AAAGGCTACGAACAGGATGTG-3 |
|  | R:5-GACTGGGTCAGGGAGAAGG-3 |
| Human- *KLF4* | F:5’-ACCTACACAAAGAGTTCCCATC-3’ |
|  | R:5’-TGTGTTTACGGTAGTGCCTG-3’ |
| Human-*ANP* | F:5-AATTTGCTGGACCATTTGGA-3 |
|  | R:5-GCTTCTTCATTCGGCTCACT-3 |
| Human-*GAPDH* | F:5-GAGTCAACGGATTTGGTCGT-3 |
|  | R:5-TTGATTTTGGAGGGATCTCG-3 |
| Human-*BNP* | F:5-AAGATGGTGCAAGGGTCTG-3 |
|  | R:5-TGTGGAATCAGAAGCAGGTG-3 |
| Human-*β-MHC* | F:5-CAGGGTGTTGACCTTGTCCT-3 |
|  | R:5-TGATCTGGAGCTGACACTGG-3 |
| Rat-Siat7A | F:5-GAGCCAAGCACAAGGGTTTC-3 |
|  | R:5-GTGGGTGTCAGGGTCACGAT-3 |
| Rat-Klf4 | F: 5-CCAGTATACATTCCGCCACAG-3 |
|  | R: 5-TCTGGGCTTCCTTTGCTAAC-3 |
| Rat-Anp | F:5-AGTGCGGTGTCCAACACAGA-3 |
|  | R:5-TCATCTTCTACCGGCATCTTCTC-3 |
| Rat-Bnp | F:5-TTCCAAGATGGCACATAGTTCAA-3 |
|  | R:5-AGCCAGGAGGTCTTCCTAAAACA-3 |
| Rat-α-sketetal actin | F:5-AGGACCTGTACGCCAACAAC-3 |
|  | R:5-ACATCTGCTGGAAGGTGGAC-3 |

**3. Results**


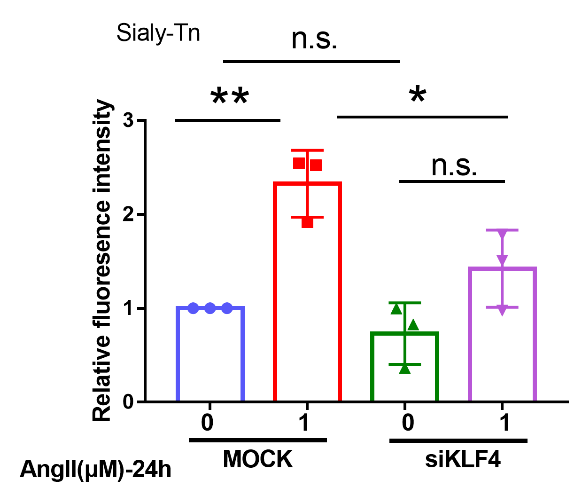


FigS1 Effects of KLF4 knockdown on expression of Sialyl-Tn in hypertrophic cardiomyocytes

The results are shown as the mean ± SD of three independent experiments. **, p < 0.01; ***, p < 0.001; ****, p < 0.0001.
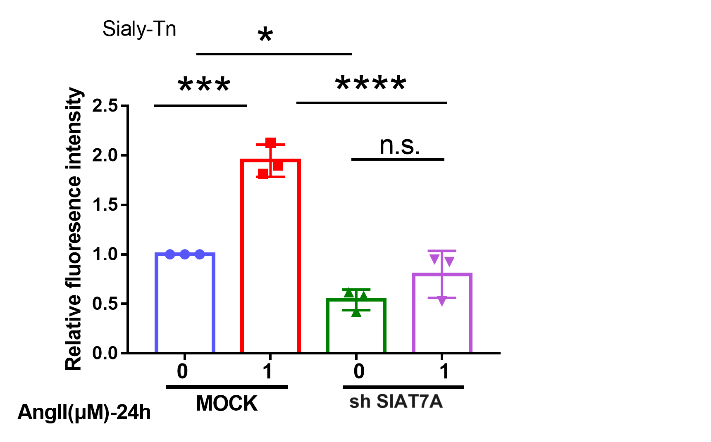


FigS2 Effects of SIAT7A knockdown on expression of Sialyl-Tn in hypertrophic cardiomyocytes

The results are shown as the mean ± SD of three independent experiments. **, p < 0.01; ***, p < 0.001; ****, p < 0.0001.


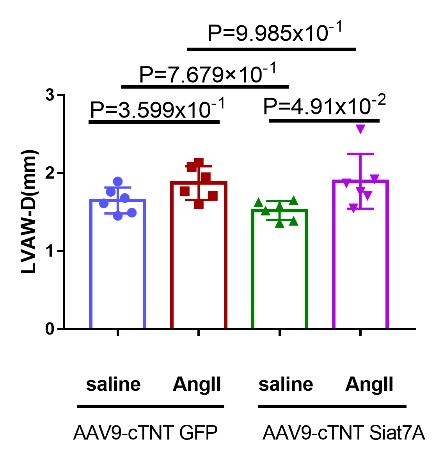


FigS3 Effect of *Siat7A* overexpression on LVAW-D of rat

Representative M-mode echocardiography of left ventricular chambers. Assessment of LVAW-D (mm) (n = 5/group). The results are shown as the mean ± SD. n.s., not significant; *, p < 0.05; **, p < 0.01; ***, p < 0.001.


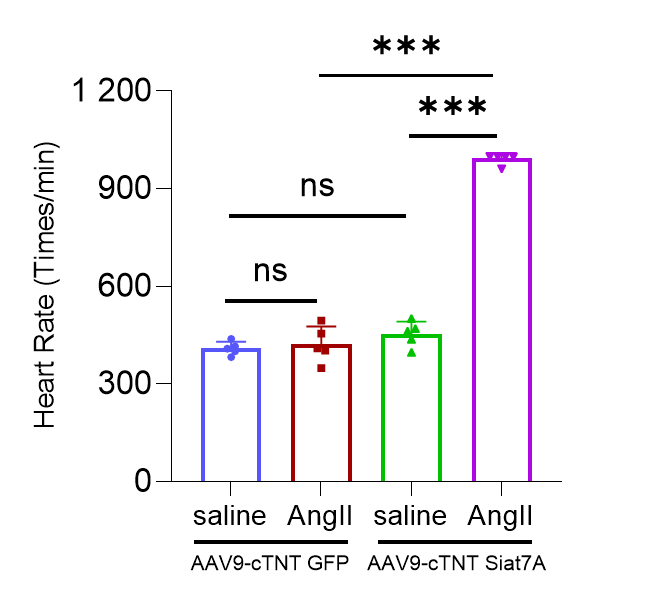


FigS4 Effect of *Siat7A* overexpression on heart rate of rat

The results are shown as the mean ± SD. of five independent experiments, n.s., not significant; *, p < 0.05; **, p < 0.01; ***, p < 0.001.


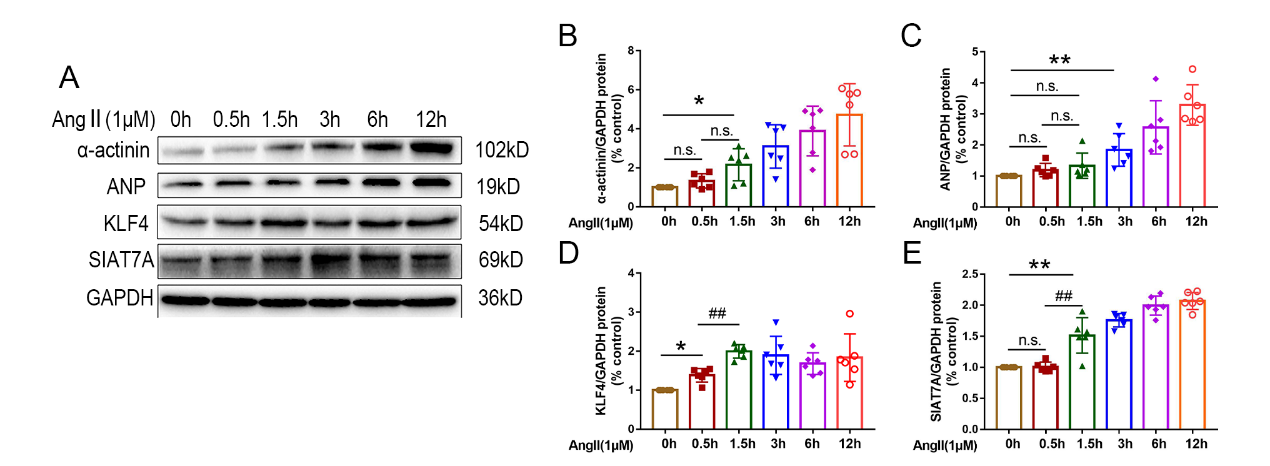


FigS5 The interaction between KLF4 and Siat7ASIAT7A during the development of myocardial hypertrophy.

The results are shown as the mean ± SD of six independent experiments. n.s., not significant; *, p < 0.05, vs 0h; **, p < 0.01, vs 0h; ^##^, p < 0.05, vs 0.5h
